# Phosphorous fertilization alleviates drought effects on *Alnus cremastogyne* by regulating its antioxidant and osmotic potential

**DOI:** 10.1038/s41598-018-24038-2

**Published:** 2018-04-04

**Authors:** Akash Tariq, Kaiwen Pan, Olusanya Abiodun Olatunji, Corina Graciano, Zilong Li, Feng Sun, Lin Zhang, Xiaogang Wu, Wenkai Chen, Dagang Song, Dan Huang, Tan Xue, Aiping Zhang

**Affiliations:** 1 CAS Key Laboratory of Mountain Ecological Restoration and Bioresource Utilization & Ecological Restoration Biodiversity Conservation Key Laboratory of Sichuan Province, Chengdu Institute of Biology, Chinese Academy of Sciences, Chengdu, 610041 People’s Republic of China; 2 University of Chinese Academy of Sciences, Beijing, 100039 People’s Republic of China; 3 Instituto de Fisiología Vegetal, Consejo Nacional de Investigaciones Científicas y Técnicas – Universidad Nacional de La Plata, Buenos Aires, Argentina

## Abstract

*Alnus cremastogyne*, a broad-leaved tree endemic to south-western China, has both commercial and restoration importance. However, little is known of its morphological, physiological and biochemical responses to drought and phosphorous (P) application. A randomized experimental design was used to investigate how drought affected *A. cremastogyne* seedlings, and the role that P applications play in these responses. Drought had significant negative effects on *A. cremastogyne* growth and metabolism, as revealed by reduced biomass (leaf, shoot and root), leaf area, stem diameter, plant height, photosynthetic rate, leaf relative water content, and photosynthetic pigments, and a weakened antioxidative defence mechanism and high lipid peroxidation level. However, the reduced leaf area and enhanced osmolyte (proline and soluble sugars) accumulation suggests drought avoidance and tolerance strategies in this tree. Applying P significantly improved the leaf relative water content and photosynthetic rate of drought-stressed seedlings, which may reflect increased anti-oxidative enzyme (superoxide dismutase, catalase and peroxidase) activities, osmolyte accumulation, soluble proteins, and decreased lipid peroxidation levels. However, P had only a slight or negligible effect on the well-watered plants. *A. cremastogyne* is sensitive to drought stress, but P facilitates and improves its metabolism primarily via biochemical and physiological rather than morphological adjustments, regardless of water availability.

## Introduction

Global climate models predict alterations in inter- and intra-annual precipitation in many regions of the world^1^. Drought is a main ecological factor limiting plant growth and development as it often leads to diminished water flux, stomata closure and decrease in carbon dioxide (CO_2_) fixation^2^. Prolonged drought can inhibit assimilation of photosynthetic CO_2_ through stomatal (e.g., stomata closure and reduced mesophyll conductance) or non-stomatal (e.g., degradation of photosynthetic pigments) factors^3,4^. CO_2_ inhibition leads to excess excitation energy and electron fluxes to O_2_, which results in photo-oxidative damage of the cell components through an over-generation of reactive oxygen species (ROS), and eventually photo-inhibition^5^. These ROS, which include superoxide ions (O_2_^•−^) and hydrogen peroxides (H_2_O_2_), form as natural products of the normal metabolism of oxygen; work as secondary messengers in redox signal transduction. However, under drought stress, an excessive generation of ROS results in substantial oxidative damage of proteins, DNA, and lipids, thereby inhibiting plant growth^6^.

Yet plants have evolved numerous protective mechanisms to protect their photosynthetic apparatus from the possible ROS-driven damage and photo-inhibition. For example, changes in the size of light-harvesting antennae can lower light energy absorption when the ability of CO_2_ assimilation declines^7^. Moreover, non-photochemical (i.e., xanthophyll cycle-mediated thermal dissipation) and photochemical (i.e., photorespiration and the water–water cycle) pathways are involved in removing excess excitation energy from the electron transport chain of photosynthesis^8^. Meanwhile, complex ROS scavenging enzymatic (superoxide dismutase [SOD], peroxidase [POD], catalase [CAT], etc.) and non-enzymatic antioxidant systems, such as low-molecular mass antioxidants (glutathione, ascorbate, carotenoids), are also involved in managing excessive energy to alleviate oxidative stress under drought conditions^9^. These enzymatic components may directly scavenge ROS or produce a non-enzymatic antioxidant. Specifically, SOD is involved in the dismutation of O_2_^•−^ into H_2_O_2_ in the mitochondrion, chloroplast, peroxisome and cytoplasm, while POD scavenges for H_2_O_2_ produced through the dismutation of O_2_^•−^ catalysed by SOD. CAT is a principal enzyme that eliminates H_2_O_2_ in the mitochondrion and microbody^10^, thus helping to ameliorate the detrimental effects of oxidative stress.

One way to sustain cell growth and to avoid cell damage from dehydration under drought stress is to actively decrease the osmotic potential. Using this strategy, water enters the cell due to a water potential gradient. In this process, plants decrease their cellular osmotic potential by accumulating more solutes (proline and soluble sugars). These compounds play crucial roles in maintaining osmotic equilibrium and protecting macromolecules, as well as membranes, thereby providing resistance against drought and cellular dehydration^6^. Specifically, the OH group of water is replaced by the hydroxyl group of sugar alcohols to maintain the hydrophilic connections with the membrane proteins and lipids. Thus, these osmolytes can support plants to sustain the strength and normal function of membranes. The most notable property of these osmolytes is that they do not affect normal metabolism of the cell. However, a response or tolerance to drought stress varies with plant species, stress intensity and growth stage^11^.

Beside the effects of drought stress for reducing cell and plant growth, stomatal conductance and photosynthetic rate, drought can depress plant growth by reducing nutrient uptake, transport and redistribution^12^. Phosphorus (P) is a major element present in plant tissues and its low mobility in soil causes its deficiency there; as a consequence, various changes in physiology, morphology and biochemistry of plants can occur depending on P availability. A majority of studies have indicated that plants decrease P uptake as soil moisture declines^13,14^. Drought can hinder P uptake by decreasing P distribution to roots, and other factors related with water relations in the affected plant^15,16^. Phosphate fertilizer is commonly applied to reduce the P deficiency in soil and to increase the drought-tolerating ability of plants. It can contribute to a controlled and proper adjustment of the physiological, morphological, and biochemical processes of a plant to promote its growth^17,18^. Despite the immense importance of P, few studies have actually assessed the effect of a limited supply of P and its application in relation to other key abiotic factors (i.e., water, CO_2_ and other nutrients) on tree physiological and ecological processes under drought stress^19,20^. Most studies have assessed the association between P fertilization and metabolic responses in non-woody drought-stressed plants. Much less attention has been paid to examining drought effects in woody species and the role of P application in mitigating drought’s negative effects.

*Alnus cremastogyne* is a fast-growing tree species endemic to south-western China. It is deciduous, thermophilic, heliophilous and tolerates poor soil, and is a non-leguminosae nitrogen-fixing plant with rhizobium colonizing its roots^21^. This tree has prospective to be a very favourable broad-leaved tree species for both commercial and vegetation restoration purposes^22^, especially when this species is planted in restoration schemes in its natural distribution area. Nevertheless, monitoring the possible threats to this species would be beneficial to prevent it from becoming threatened. It has been suggested that *A. cremastogyne* is very sensitive to drought stress and that it has low drought resistant potential under short-term stress (<18 days)^23^. However, short-term experiments cannot confirm its full response to longer bouts with drought stress, which more frequently occurs under field conditions. Under long-term stress, plants can develop acclimation strategies that are not readily observed under imposed states of sudden, short-term stress. We still lack sufficient understanding of longer periods of drought affects the growth and metabolism of *A. cremastogyne*. Moreover, to our best knowledge, there is no published research examining the role of P fertilization in alleviating drought effects in *A. cremastogyne*. To fill this gap, several questions should be addressed: What kind of morphological, physiological and biochemical strategies does this tree species have to tolerate drought stress? How does P-fertilizer application possibly enhance this tree species tolerance to drought stress?

The present study was therefore designed with the following objectives: (1) To evaluate the morphological, physiological and biochemical responses of *A. cremastogyne* to drought, by considering the effect of drought at both the leaf level (photosynthetic capacity, water status and antioxidant metabolism) and whole plant level (growth and dry mass partitioning), and (2) To examine whether P application can mitigate the negative impact of drought by improving this species drought tolerance capacity, via changes at the leaf or whole plant level, or both. To achieve these objectives we investigated plant growth and dry mass partitioning, water status, gas exchange, chlorophyll florescence, ROS production rate, antioxidant enzyme activities and several biochemical parameters. This study clarifies our understanding of *A. cremastogyne* physiology and contributes to the proper management of this tree species in its native forest and in restoration programs in the face of climate change.

## Results

### Morphological traits

Overall, a strong reduction was observed in the morphological traits of drought-stressed plants when compared with their well-watered counterparts (Table 1). Irrespective of the P application, there was a significant reduction in leaf biomass (38.29%), shoot biomass (65.73%), leaf area (50.32%) and stem diameter (24.88%) of *A. cremastogyne* under drought stress compared with well-watered conditions. P application slightly increased the leaf biomass, shoot biomass, root biomass and height under drought stress in comparison with the well-watered plants, but these differences were not significant. Similarly, P application did not have any significant effects on the morphological traits of the well-watered plants. The ration of leaf:root biomass was lower in the plants well-watered (c. 0.47) than in those under drought stress (c. 0.65), irrespective of the P application. Therefore, P fertilization did not change dry mass partitioning in this tree species’ seedlings.

**Table 1:** Changes in growth and dry mass partitioning in response to P application under well-watered and drought conditions.

Traits	Well-watered		Water-stressed	
	−P	+P	−P	+P
Leaf biomass (g)	4.7 ± 0.28a	5.24 ± 0.48a	2.9 ± 0.24b	3.53 ± 0.3b
Shoot biomass (g)	23.96 ± 4.24a	22.57 ± 3.64a	8.21 ± 0.92b	10.56 ± 1.31b
Root biomass (g)	9.83 ± 0.94a	11.31 ± 0.47a	4.58 ± 0.31b	5.27 ± 0.64b
Leaf area (cm^2^)	41.53 ± 1.91a	44.45 ± 2.31a	20.63 ± 3.09b	17.98 ± 1.22b
Height (cm)	72.33 ± 4.33ab	77.66 ± 4.67a	54.33 ± 5.17c	66.16 ± 5.78bc
Stem diameter (mm)	6.68 ± 0.23a	6.43 ± 0.22a	5.2 ± 0.3b	5.28 ± 0.54b

Means followed by different letters indicate significant differences (*P* ≤ 0.05) among the four treatments according to Duncan’s test. Values are means ± SE.

### Leaf relative water content, gas exchange, and chlorophyll fluorescence

Leaf relative water content (LRWC) of *A. cremastogyne* significantly decreased under drought stress by 32.61% when compared with the well-watered treatment, irrespective of the P application (Table 2). The P application significantly increased LRWC under drought, by 25.09%, when compared with its counterpart (−P); however, it had no effect on the well-watered plants. Drought significantly decreased the net CO_2_ assimilation rate (*P*_n_) (78.14%) in comparison with the well-watered plants; however, the P application significantly increased *P*_n_ under both watering treatments. Moreover, there was a significant reduction in the intercellular CO_2_ concentration (*C*_i_), stomatal conductance (*G*_s)_, transpiration rate (*E*) and maximum quantum efficiency of photosystem II (*F*_*v*_*/F*_*m*_) in the plants drought-stressed when compared with those well-watered, irrespective of the P application. However, P application did slightly increase the above mentioned leaf physiological traits, though no significant differences were detected.

**Table 2:** Changes in leaf relative water content, and the photosynthetic and chlorophyll fluorescence parameters in response to P application under well-watered and drought conditions.

Traits	Well-watered		Water-stressed	
	−P	+ P	−P	+ P
LRWC (%)	72.06 ± 3.7a	73.93 ± 2.29a	47.3 ± 1.49c	59.17 ± 2.77b
*P*_n_ (µmol m^−2^ s^−1^)	6.68 ± 0.59b	8.83 ± 0.6a	1.46 ± 0.31d	2.97 ± 0.21c
*C*_i_ (µmol mol^−1^)	223.79 ± 8.9a	236.43 ± 14.5a	158.49 ± 12.14b	196.05 ± 11.31ab
*G*_s_ (mol m^−2^ s^−1^)	0.08 ± 0.01ab	0.09 ± 0.01a	0.04 ± 0.01b	0.06 ± 0.01ab
*E* (mmol m^−2^ s^−1^)	2.69 ± 0.24a	3.29 ± 0.39a	1.04 ± 0.26b	1.28 ± 0.2b
*F*_v_ */F*_m_	0.76 ± 0.06a	0.79 ± 0.03a	0.55 ± 0.05b	0.53 ± 0.06b

Means followed by different letters indicate significant differences (*P* ≤ 0.05) among the four treatments according to Duncan’s test. Values are means ± SE.

### Photosynthetic pigments

**Figure 1:**
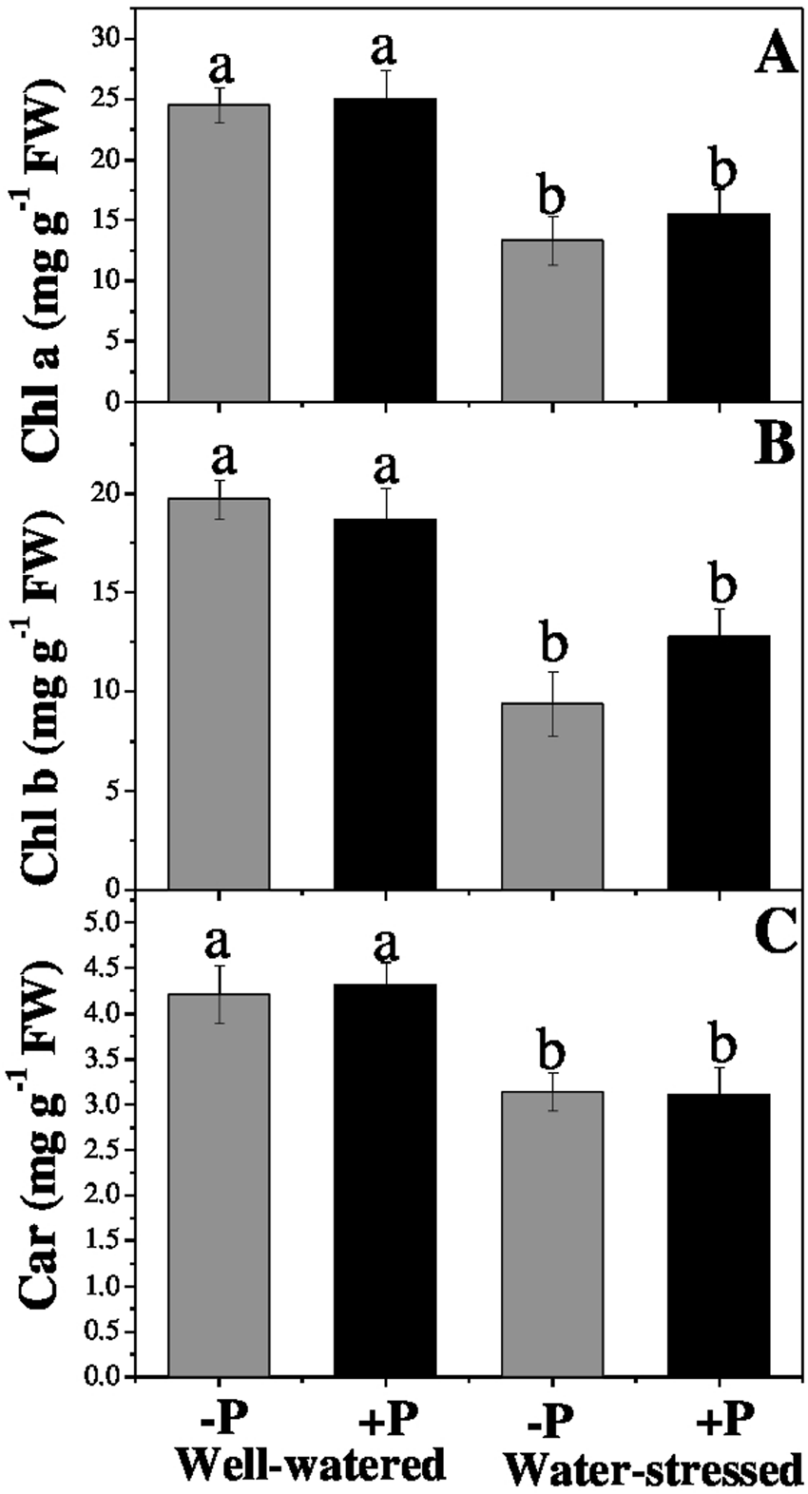
Changes in (**A**) chlorophyll a, (**B**) chlorophyll b, and (**C**) carotenoids in response to P application under well-watered and drought conditions. Means followed by different letters indicate significant differences (*P* ≤ 0.05) among the four treatments according to Duncan’s test. Bars show means ± SE.

Photosynthetic pigments concentrations chlorophyll *a* (Chl *a*), chlorophyll *b* (Chl *b*), and carotenoids (Car) were significantly lower in the plants drought-stressed than in those well-watered, regardless of the P application (Fig. 1). Phosphorous fertilization did not significantly increase the Chl *a* and Chl *b* concentrations under drought stress when compared with the well-watered treatment. However, the P application apparently had no effect on Car concentration in the drought-stressed plants.

### Antioxidative defence system

**Figure 2:**
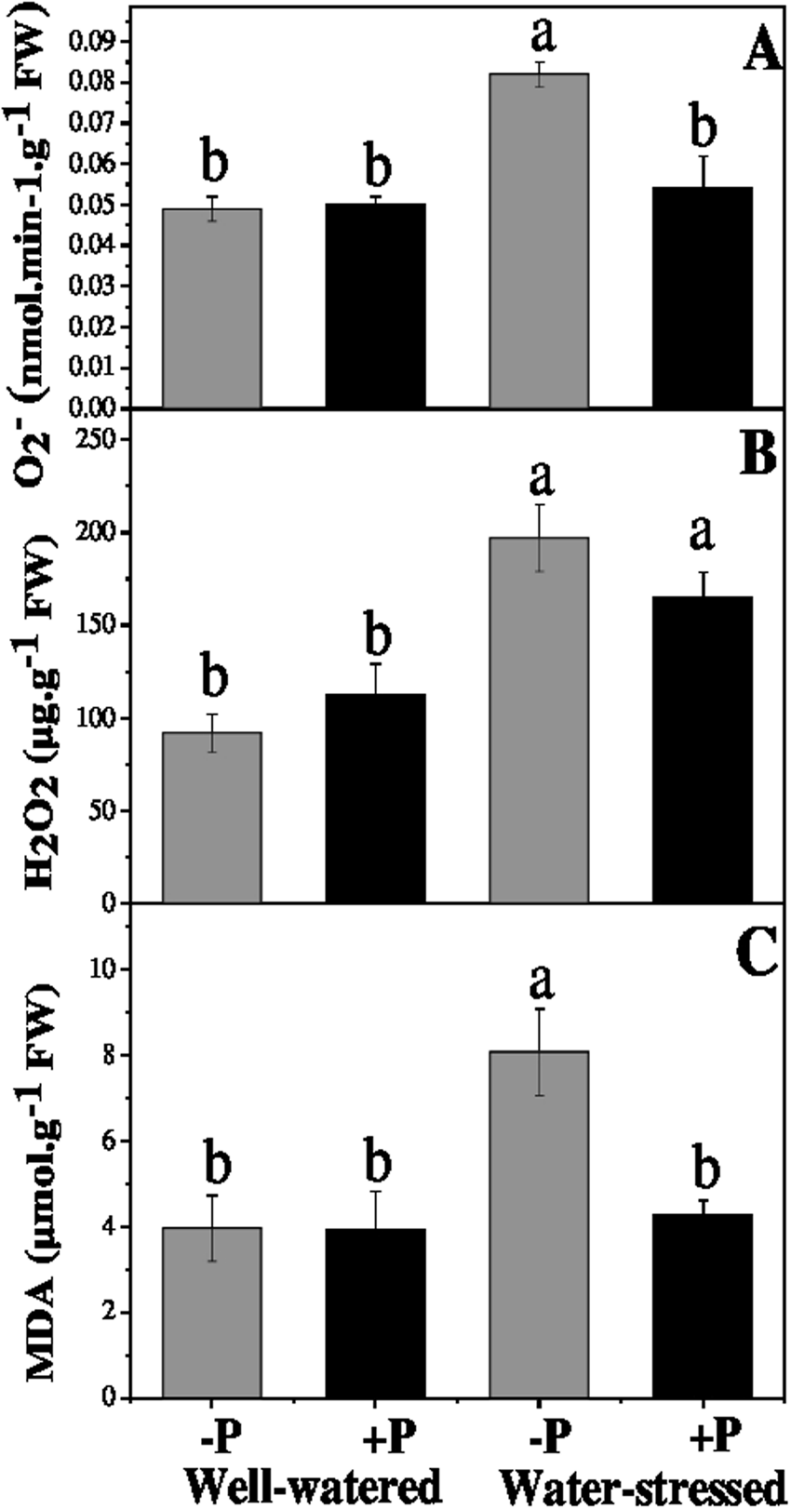
Changes in (**A**) lipid peroxidation superoxide anion, (**B**) hydrogen peroxide, and (**C**) malondialdehyde in response to P application under well-watered and drought conditions. Means followed by different letters indicate significant differences (*P* ≤ 0.05) among the four treatments according to Duncan’s test. Bars show means ± SE.

Lipid peroxidation was significantly higher in the drought-stressed plants than in the well-watered non-fertilized plants (Fig. 2). P application significantly decreased malondialdehyde (MDA) contents by 47.09% in plants under drought stress in comparison with their non-fertilized counterparts. Moreover, the H_2_O_2_ and O_2_^•−^ production rate significantly accelerated in the drought-stressed plants when compared with the well-watered non-fertilized plants. However, the P application significantly decreased the O_2_^•−^ (34.14%) level in plants under drought stress when compared with their non-fertilized counterparts, while it slightly decreased the H_2_O_2_ (16.27%) level (though not significantly).

**Figure 3:**
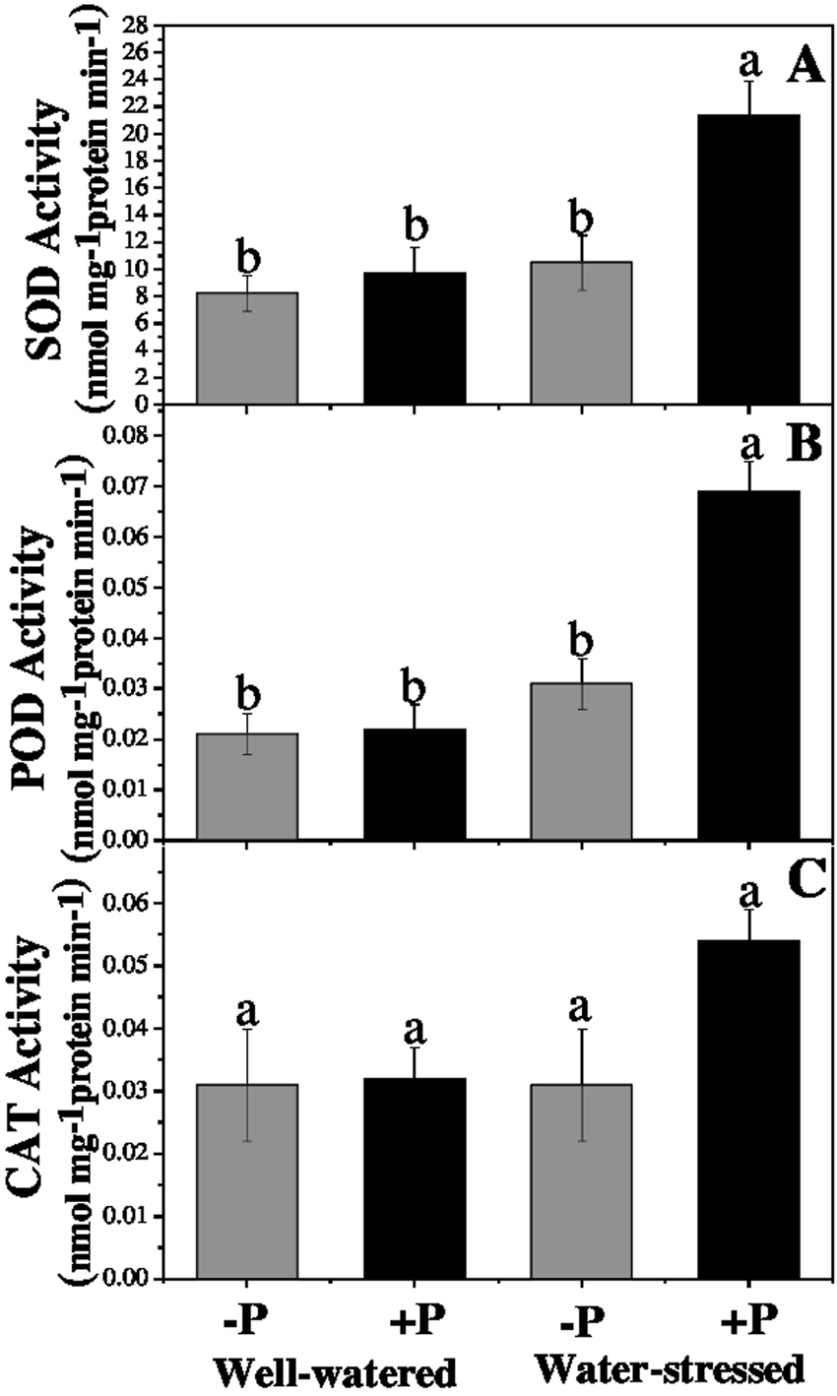
Changes in (**A**) superoxide dismutase, (**B**) peroxidase, and (**C**) catalase in response to P application under well-watered and drought conditions. Means followed by different letters indicate significant differences (*P* ≤ 0.05) among the four treatments according to Duncan’s test. Bars show means ± SE.

Under drought stress, the activity of SOD, POD, and CAT was similar to that in the well-watered non-fertilized plants. However, applying P significantly increased both POD (22.58%) and SOD (103.81%) activities under drought-stressed plants when compared with their non-fertilized counterparts. The P application also increased CAT activity in the drought-stressed plants, by 50%, but this difference was not significant (Fig. 3).

### Osmolytes and soluble proteins

**Figure 4:**
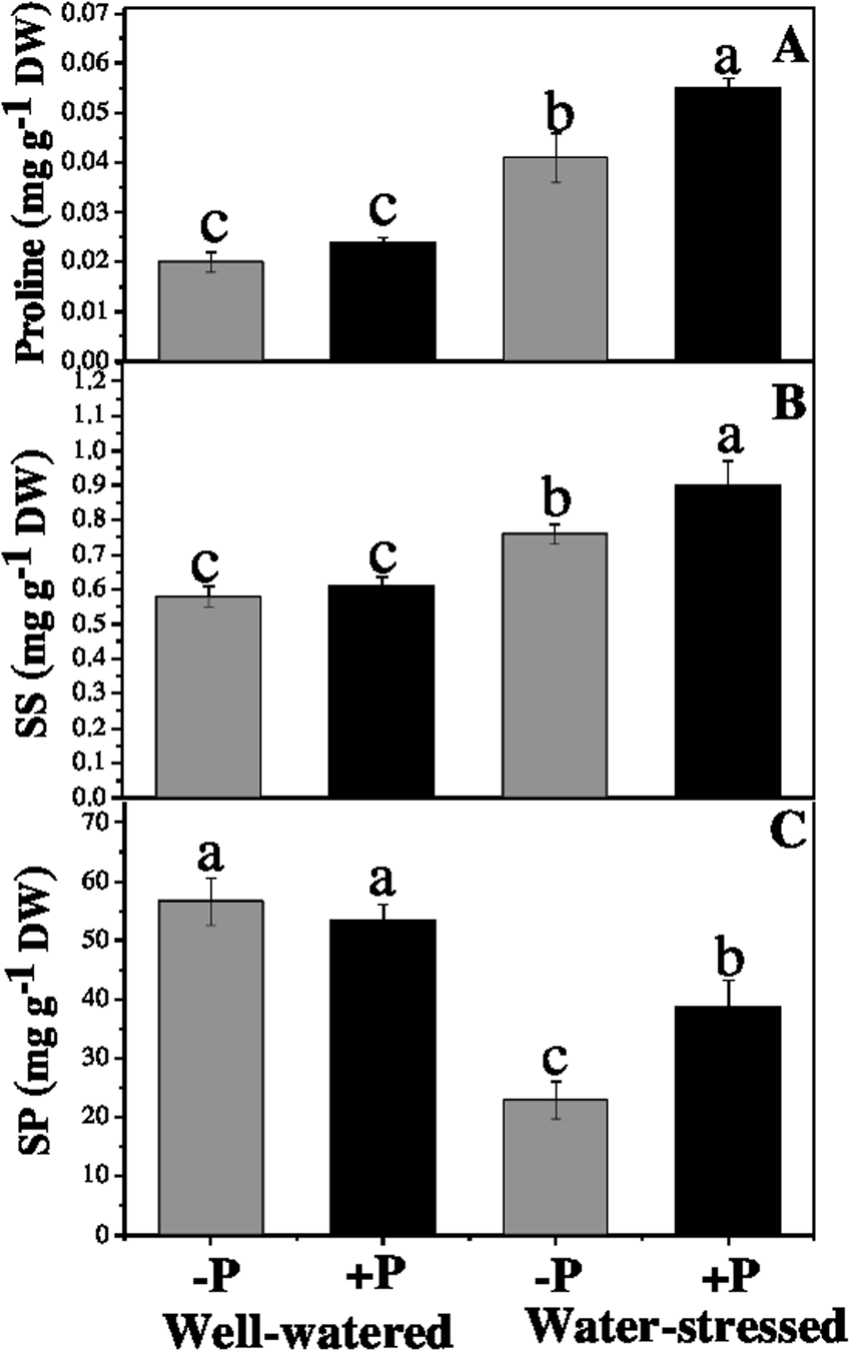
Changes in (**A**) proline, (**B**) soluble sugars, and (**C**) soluble proteins in response to P application under well-watered and drought conditions. Means followed by different letters indicate significant differences (*P* ≤ 0.05) among the four treatments according to Duncan’s test. Bars are means ± SE.

Concentrations of both proline and soluble sugars (SS) were higher, whereas the soluble proteins (SP) was lower, in the plants drought-stressed when compared with those well-watered (Fig. 4). P application under drought significantly increased the proline (34.41%), SS (18.4%) and SP (68.3%) concentrations in the plants when compared with their non-fertilized counterparts. However, the P application had no significant effects on the above biochemical traits of the well-watered plants.

## Discussion

### Plant growth

Drought stress is the primary abiotic threat to plant growth around the world, especially under arid and semiarid conditions. Permanent or temporary drought events severely hamper plant growth and development and also restricts nutrient uptake by plant organs more so than any other environmental factor^24^. Our result showed that biomass and growth of *A. cremastogyne* was significantly reduced under drought stress when compared with well-watered growing conditions, which may be explained by associated leaf area and stem diameter reductions (Table 1). A reduction in leaf area is generally ascribed to the suppression of leaf expansion, as cells lose the turgidity necessary for this process and the declining photosynthetic rate diminishes the availability of photoassimilates to build new cells^25^. However, some plant species may reduce their leaf area size to reduce water loss through transpiration, or to enhance proportionally their root water absorption capacity. Hu *et al*.^23^ also suggested that *A. cremastogyne* adopts a drought avoidance strategy by reducing its individual leaf sizes as well as by shedding leaves (defoliation). Hence, the reduction in *A. cremastogyne* growth and biomass may be part of seedlings enacting this drought avoiding strategy, as well as being driven by declines in photosynthetic rate that we found (Table 2). Other possible reasons for reduced growth and biomass in drought-stressed plants is a water deficiency that interrupts water flow from soil to the xylem and surrounding cells, which decreases the mobility of available ions, nutrient availabilities and soil microbial activities^26,27^.

Our results also showed that P application did not enhance growth and biomass partitioning of the well-watered *A. cremastogyne* plants. An explanation for this result is that the already available P in the soil was sufficient to meet the functional requirements of plants during their establishment. Under drought stress conditions, applying P did not improve growth and biomass partitioning of *A. cremastogyne*, perhaps because its diffusion rate and movement towards the root organs slows under drought stress. Our findings contrast with several researches that reported higher biomass and growth rate in P-fertilized plants, but nonetheless support other studies finding non-significant effects of P fertilization on plant growth parameters^19,28,29^. These varied and inconsistent responses of different plant species to P fertilization in terms of their growth are perhaps not so surprising: species identity, soil physical properties, nutrient availability and their interactions, among other factors, likely control how individual plants respond to an increased concentration of a particular nutrient in the soil.

### LRWC, gas exchange, and chlorophyll fluorescence

Water relations in plants are important for maintaining their normal growth and metabolism when challenged by drought. The ability to maintain a high water potential is one of the most imperative challenges faced by many terrestrial plant species. Drought stress affects hydraulic conductivity of plants, driving changes in their shoots and the loss of turgor in their leaves^30^. LRWC is therefore considered an effective and reliable indicator for measuring the drought tolerance of plants. Regardless of the P application, LRWC was significantly lower in the plants drought-stressed than those well-watered, suggesting a low tolerance to drought stress in *A. cremastogyne*. Previous studies also suggested the same physiological response in different tree species^31,32^. Applying P significantly increased the LRWC under drought stress but not so in the well-watered plants (Table 2), which may be associated with the higher accumulation of osmolytes under P fertilization (Fig. 4) improving its osmotic adjustment. Our findings are in line with prior studies suggesting an improved LRWC in drought-stressed plants under P fertilization^33,34^. However, our findings contrast with several studies that suggested non-significant effects of P application on LRWC for different plant species under drought^19,20^. This disagreement may reflect the interspecific differences in physiological, biochemical and molecular mechanisms of the studied plants, such as their gene expression and protein assimilation.

Drought stress negatively affected *P*_n_ due to reductions in *G*_s_ and *C*i, as well from damage to the photosynthetic apparatus as shown by the decrease in *F*_v_*/F*_m_. Reduced stomatal conductance may serve as another drought avoiding strategy of *A. cremastogyne*, enabling it to reduce its transpiration rate to better tolerate the physiological stress imposed by drought. The reason for a low stomatal conductance, or stomatal closure, may be a drought stress-induced synthesis of high amounts of abscisic acid (ABA) in the roots, which is then transported to the shoots. ABA stimulates the K+ ion efflux from the guard cells, resulting in loss of turgor pressure, which decreases *G*_s_^35^. This reduction in *G*_s_ limits *C*_i_ and leads to a decreased *P*_n_, as shown by our results (Table 2), a process that might be driven by declines in Rubisco activity^36^. However, P fertilization significantly increased *P*_n_ in the drought-stressed plants without affecting *G*_s_, *C*_i_ or *F*_v_*/F*_m_. These results are consistent with other studies reporting an improved photosynthetic rate in different drought-stressed plant species that were P-fertilized^19,37^. Thus, our findings suggest that P application can augment the ability of *A. cremastogyne* seedling to tolerate drought stress (as indicated by their increased LRWC and *P*_n_).

### Photosynthetic pigments

Photosynthetic pigments are essential for plants to harvest light; however, a decline in their concentrations can limit the photosynthetic rate of leaves and primary production^38^. In our study, the concentrations of photosynthetic pigments were significantly lower in the drought-stressed plants compared with the well-watered plants, which may be another reason for the decreased *F*_v_/*F*_m_ and *P*_n_. This result clearly indicates that drought stress impairs the *A. cremastogyne* photosystem in leaves by degrading their photosynthetic pigments. Similar findings of drought having negative effects on chloroplast pigments have been reported in other studies^39,40^. Phosphorous application slightly improved the photosynthetic pigments’ concentration, but no significant differences were observed. These findings provide an interesting insight: P fertilization significantly improves *P*_n_ in drought-stressed plants but only slightly improves the pigments’ concentrations, possibly for efficient regulation of the available amount of light. Our results agree and disagree with several previous studies, which may be due to the differences in duration and severity of drought investigated by them^18,37,38^.

### ROS production and antioxidative enzymes

Under stressful conditions, MDA accumulation leads to the damage of the cell membrane in plants^41^. MDA is measured as a suitable indicator for membrane lipid peroxidation. Our results showed that MDA contents were significantly higher in drought-stressed plants than in the well-watered ones due to a significant increase in ROS production. In plant cells, ROS (O_2_^•−^ and H_2_O_2_) act as signalling molecules; however, their excessive production can trigger fragmentation of DNA, proteins degradation, lipid peroxidation, and may even cause cell death^9^. The ROS level was significantly higher in drought-stressed than in well-watered plants when both were not fertilized. However, among the P-fertilized plants, ROS and MDA were similar between those well-watered and under drought-stressed; hence, fertilization with P avoids an ROS increment under drought stress. An increase in the ROS level under drought conditions was attributed to a reduced photosynthetic rate, as shown in our results (Table 2) and also by previous studies^42^. The induction of antioxidant enzyme activities is a general defence mechanism that plants use against drought stress; it helps them to overcome oxidative stress and associated cell damage from it. Key antioxidative (SOD, POD and CAT) enzyme activities were consistently found to be higher in drought-stressed plants fertilized with P than in those non-fertilized. Therefore, under drought stress, applying P improves the antioxidant defence mechanism of *A. cremastogyne* seedling, which was efficient enough to remove ROS and decrease the MDA level with respect to the non-fertilized drought stress plants. Interestingly, P fertilization significantly reduced MDA accumulation and O_2_^•–^ production (Fig. 3) due to significantly increased SOD and POD activities (Fig. 4). In sum, our findings indicate that *A. cremastogyne*’s antioxidative defence mechanism has a weak efficiency when responding to drought-induced damage; however, via P fertilization, its efficiency is strengthened sufficiently to mitigate the negative effects of drought.

### Soluble sugars, proline and soluble proteins

Plants typically accumulate different osmolytes in the cell’s cytosol for increasing osmotic potential in order to tolerate drought stress, since it improves or maintains turgidity and the continuation of plant growth processes. Moreover, these osmolytes also detoxify ROS, stabilize membrane and protect macromolecules^43^. Our results showed that plants under drought stress accumulated more proline and SS than did the ones well-watered. However, the SP concentration was lower in plants drought-stressed than in those well-watered. Possible reasons for this result include an associated increased function of protease enzymes, proteolysis or decreased protein synthesis, as well as a lower *P*_n_ (i.e., less carbon to build any metabolite), in drought-stressed plants. Moreover, the P fertilization significantly increased the SS, proline and SP concentrations in the drought-stressed plants when compared with their non-fertilized counterparts. Shubra *et al*.^31^ similarly reported an increased SS concentration in response to P application under drought conditions, which may have been caused by the reduction in of normal SS transport, utilization and distribution during water stress, or the hydrolysis of starch. Al-Karaki *et al*.^44^ also observed higher accumulation of proline in P-fertilized sorghum plants, while Azcon *et al*.^45^ also found a positive role of P application on the reduction and assimilation of nitrogenous compounds. Our findings clearly indicate that *A. cremastogyne* can maintain the osmotic potential of its cells under drought stress; however, P fertilization further enhances its osmotic potential and LRWC, which further improve its drought tolerance.

Drought stress had significant negative effects on the growth and metabolism of *A. cremastogyne* seedlings as indicated by their reduced biomass, leaf relative water content, photosynthetic rate, pigment concentrations, higher malondialdehyde level and inefficient antioxidative defence system. However, the reduced leaf area and higher osmolytes accumulation also reduced water losses and maintained the osmotic potential of the cells, suggesting it may serve as drought-avoidance or tolerant strategy. Phosphorous application had negligible effects on the morphological traits of this tree species when its seedlings were either drought-stressed or well-watered. In the former, however, P fertilization significantly improved the antioxidative enzymes activities, osmolytes accumulation, and also decreased the malondialdehyde level, so that their leaf relative water content and photosynthetic rate were improved. These results reveal that phosphorous fertilization facilitates *A. cremastogyne* under drought stress, mostly through physiological and biochemical adjustments at leaf level rather than changes made at the whole plant level in terms of growth or dry mass partitioning. Nonetheless, it is now imperative to investigate the underlying biochemical, physiological and molecular mechanisms, as well as the possible role of different levels of P fertilization under drought stress, in order to design effective measures for the proper management of this valuable tree species.

## Materials and Methods

### Plant collection and experimental design

The experiment was carried out at the Centre for Ecological Studies at the Chinese Academy of Sciences, Sichuan, in south-west China. Healthy and uniform, 2-year-old seedlings of *A. cremastogyne* were collected from Sichuan Agricultural University, Sichuan province, and transferred into 10-L plastic pots containing approximately 4 kg of homogenized topsoil (pH 7.3; total nitrogen, 0.19%; carbon, 2.67%). These pots were organized in a greenhouse (temperature range: 18–32 °C; relative humidity range 50–85%) following a complete randomized block design and watered regularly. Availability of light inside the greenhouse was kept homogeneous, and there was 6–9% reduction in direct sunlight. After growing for 2 months in the greenhouse, the seedlings were assigned to three replicates of four treatments for 90 days: two water regimes (well-watered vs. water-stressed) and two levels of P fertilization (with vs. without P fertilization). First, total and available P in soil was measured before applying the treatments, and found to be 0.89 g kg^−1^ and 27.6 mg kg^−1^, respectively. Extraction of available P was done with 0.5 M of NaHCO_3_ (pH 8.2) according to Olsen and Sommers^46^ and colorimetric measurement was done by the molybdate-ascorbic acid as described by Murphy and Riley^47^. Weight method was used to calculate soil relative water content (SRWC) of the two water treatments (control: 80–85%; severe drought: 30–35%)^48^. The pots were weighed daily and watered up to their respective target SRWC, by replacing the amount of water transpired and evaporated. SRWC was expressed as follows:

1 $${\rm{SRWC}}=[({W}_{{\rm{soil}}}-{W}_{{\rm{pot}}}-D{W}_{{\rm{soil}}})/({W}_{{\rm{FC}}}-{W}_{{\rm{pot}}}-D{W}_{{\rm{soil}}})]\ast 100$$

where *W*_soil_ is the current soil weight (soil + pot + water), *W*_pot_ is the weight of the empty pot, *DW*_soil_ is the dry soil weight, and *W*_FC_ is the soil weight at field capacity (soil + pot + water).

Phosphorous fertilization was supplied as sodium di-hydrogen phosphate (NaH_2_PO_4_, 25.5% P), with the dose consisting of 129.3 mg P mixed in 200 mL of water per pot, applied every 30 days (i.e., three times for the entire experiment). To avoid systematic error produced by fluctuations in the local environmental conditions, the pots were rotated after every 5 days during the experiment. Plant samples were collected at the end of the experiment. From each plant, the upper fully-expanded leaves were used for all the physiological and biochemical determinations.

### Analysis of growth and biomass

Plant height (cm), stem diameter (mm) and leaf area (cm^2^) were quantified in a standard way by using a measuring tape, electronic calipers, and leaf area meter (CI 202, USA), respectively. After removing the experimental plants from the soil, their roots, shoots and leaves of were separated and a subset of them oven-dried at 70 °C for 24 h to obtain their dry weights.

### Determination of leaf relative water content

From each pot fully expanded leaves were collected as a single sample and their fresh weight (FW) measured. Then, the samples were immediately dipped into distilled water, in the dark at a 4 °C temperature. After 4 h, leaves were weighed to obtain their turgor weight (TW) and then put in an oven for 24 h at 70 °C to determine their dry weight (DW).

The following equation was used to calculate the LRWC of the samples:

2 $${\rm{LRWC}}=[({\rm{FW}}-{\rm{DW}})/({\rm{TW}}-{\rm{DW}})]\times \mathrm{100} \% $$

### Gas exchange and chlorophyll fluorescence measurements

The net CO_2_ assimilation rate (*P*_n_), stomatal conductance (*G*_s_), intercellular CO_2_ concentration (*C*_i_) and transpiration rate (*E*) were measured from fully expanded leaves at similar development stages with a portable open-flow gas exchange system (LI-6400, LI-COR Inc., USA) during the late morning (9:00–11:00 h). The relative humidity of air, CO_2_ concentration and photon flux density were respectively maintained at 60–70%, 380 µmol mol^−1^ and 800 µmol m^−2^ s^−1^ in all cases. The maximum quantum efficiency of photosystem II (*F*_v_*/F*_m_) was measured, from the same leaves as above, with a portable pulse amplitude modulated fluorometer (PAM^−2^ 100, Walz, Effeltrich, Germany). The leaves were dark-adapted with clips for 20 mins. Then, a saturation pulse of 8000 µmol m^−2^ s^−1^ was applied for 0.8 s, and the maximal fluorescence yields (*F*_m_) and the intrinsic quantum efficiency of photosystem II (PSII) photochemistry (*F*_v_/*F*_m_) were recorded.

### Determination of photosynthetic pigments

Extraction of chlorophyll a (Chl *a*), chlorophyll b (Chl *b*) and carotenoids (Car) was performed using 2 g of fresh leaf with 5 mL of 100% acetone. The samples were then subjected to dark condition at room temperature for 36 h and their respective absorbance was measured at 662, 645 and 470 spectrophotometrically. Following equation was used for the determination of pigments concentrations: Chl a = 11.75A662 − 2.35A645; Chl b = 18.61A645 − 3.96A662; Car = (1000A470 − 2.27 Chl a − 81.40 Chl b)/227^49^.

### Determination of soluble sugars (SS), proline and soluble proteins (SP)

Leaves (0.2 g DW) were extracted three times with 6 mL of 80% ethanol at 80 °C for 30 min. The resulting supernatant was analysed for soluble sugars (SS) following the anthrone method^50^. Proline was extracted with 2 mL of 10% acetic acid and 5 mL of 3% sulfosalicylic acid. The resulting supernatants were analysed according to the method described by Liu *et al*.^49^ Soluble proteins (SP) concentrations were determined using Bradford G-250 reagent.

### Determination of ROS and lipid peroxidation

We homogenized the fresh leaves (0.2 g) using 2 mL of 65 mM phosphate buffer (pH 7.8) for the measurement of superoxide anion (O_2_^•−^) production rate and centrifuged at 5000 g for 10 min^51^. The production rate was measured by observing the nitrite formation from hydroxylamine in the presence of O_2_^•−^. The composition of incubation mixture was, 0.9 mL of 65-mM phosphate buffer (pH 7.8), 0.1 mL of 10 mM hydroxylammonium chloride and 1 mL of supernatant. After 20 min incubation at 25 °C, sulphanilamide (17 mM) and α-naphthylamine (7 mM) were added to the incubation mixture, which was then kept at 25 °C for 20 min. Ethyl ether was added in the same volume and centrifugation was performed at 1500 g for 5 min. The absorbance wavelength used for the aqueous solution was 530 nm.

Hydrogen peroxide (H_2_O_2_) determination was done by content was determined by observing the absorbance of the titanium-peroxide complex at 410 nm^52^. We homogenized fresh leaves (0.2 g) using 5 mL of acetone and centrifuged at 3000 g for 10 min. The reactive mixture contained 0.1 mL of a titanium reagent (50 μL of 20%-titanium tetrachloride in concentrated HCl), 0.2 mL of ammonia and 1 mL of supernatant; this was then centrifuged at 3000 g for 10 min. The resultant precipitate was washed for five times using acetone and centrifuged at 10 000 g for 5 min. The ensuing precipitate was then solubilized in 3 mL of 1-M H_2_SO_4_ and its absorbance read at 410 nm.

Lipid peroxidation was estimated by measuring the malondialdehyde (MDA) content, according to the thiobarbituric acid (TBA) test, at 450, 532 and 600 nm^53^. For the MDA assay, 0.25 g of fresh leaves were ground in 5 ml of 1% trichloroacetic acid (TCA) and centrifuged at 5000 g for 10 min in a refrigerated centrifuge, then 1 ml of the supernatant was added to 4 ml of 20% TCA (containing 0.5%-thiobarbituric acid). The mixture was heated at 95 °C for 30 min, quickly cooled in an ice bath, and then its absorbance read with a spectrophotometer at 450, 532, and 600 nm^53^. The MDA concentration was calculated using the following equation:

3 $${\rm{MDA}}\,({\rm{mol}}\,{{\rm{g}}}^{-{\rm{1}}}\,{\rm{FW}})={\rm{6.45}}({\rm{OD532}}-{\rm{OD600}})-{\rm{0}}{\rm{.56OD450}}$$

### Determination of antioxidant enzyme activities

To extract the antioxidant enzymes, fresh leaves (0.1 g) were ground in a mortar containing 5 ml of extraction buffer (50 mM Tris–HCl [pH 7.0], 0.1 mM EDTA, 1 mM AsA, 1 mM dithiothreitol and 5 mM MgCl_2_). The resultant homogenates were centrifuged at 10,000 *g* for 15 min at 4 °C, and the supernatants used to measure the activity of antioxidant enzymes. Superoxide dismutase (SOD) activity was determined by quantifying the inhibition of the photochemical reduction of nitroblue tetrazolium (NBT)^54^. The composition of the reaction mixture (3 ml) was 50 mM of Tris–HCl (pH 7.8), 13.37 of mM methionine, 0.1 mM of NBT, 0.1 mM of riboflavin, 0.1 mM of EDTA and 0.1 ml of the enzyme extract. One unit of enzyme activity was defined as the amount of enzyme resulting in 50% inhibition of the photochemical reduction of NBT at 560 nm. Catalase (CAT) and peroxidase (POD) activities were assessed following the methods of Fu and Huang^54^. CAT activity was determined by measuring the reduction in the absorbance of H_2_O_2_ at 240 nm. The composition of the reaction mixture (3 ml) was 50 mM of Tris–HCl (pH 7.0), 0.1 mM of EDTA, 12.5 mM of H_2_O_2_ and 0.1 ml of the enzyme extract. One unit of enzyme activity was defined as a 0.01-change in the absorbance at 240 nm per min. POD activity was assessed by measuring the increase in absorbance at 470 nm due to guaiacol oxidation. The reaction mixture was composed of 50 mM Tris–HCl (pH 7.0), 10 mM of guaiacol, 5 mM of H_2_O_2_ and 0.1 ml of the enzyme extract. One unit of POD activity was defined as an absorbance change of 0.1 units per min.

### Statistical analysis

All the measurements were repeated three times, and data organized in Microsoft Excel 2007 and presented as mean values ± SE. We used SPSS v16.0 (SPSS Inc., 2007) to perform the one-way analysis of variance (ANOVA) on the data. Duncan’s multiple range test, with an alpha level of 0.05 for significance, was used to make pairwise comparisons of the mean values for a given response variable. Before fitting the ANOVAs, the data were checked for normality and homogeneity of variances. The tool Origin pro v8.5 was used to draw the figure graphics, all of which show bars as the mean ± SE.

## References

[1] Melillo, J. M., Richmond, T. C. & Yohe, G. W. Yohe, Eds: Climate Change Impacts in the United States: The Third National Climate Assessment. U.S. Global Change Research Program, **841** pp. 10.7930/J0Z31WJ2.2014 (2014)

[2] Mahajan S, Tuteja N (2005). Cold, salinity and drought stresses: an overview. Arch. Biochem. Biophys..

[3] Cornic G, Fresneau C (2002). Photosynthetic carbon reduction and carbon oxidation cycles are the main electron sinks for photosystem II activity during a mild drought. Ann. Bot..

[4] Flexas J, Bota J, Loreto F, Cornic G, Sharkey TD (2004). Diffusive and metabolic limitations to photosynthesis under drought and salinity in C3 plants. Plant Biol..

[5] Parvaiz A, Satyawati S (2008). Salt stress and phyto-biochemical responses of plants-a review. Plant Soil Environ..

[6] Tariq A (2017). Phosphorous application improves drought tolerance of *Phoebe zhennan*. Front. Plant. Sci..

[7] Niyogi KK (1999). Photoprotection revisited: genetic and molecular approaches. Annu. Rev. Plant Physiol. Plant Mol. Biol..

[8] Badger MR, Takahashi S (2011). Photoprotection in plants: a new light on photosystem II damage. Trends. Plant. Sci..

[9] Apel K, Hirt H (2004). Reactive oxygen species: metabolism, oxidative stress, and signal transduction. Annu. Rev. Plant Biol..

[10] Shigeoka S (2002). Regulation and function of ascorbate peroxidase isoenzymes. J. Exp. Bot..

[11] Demirevska K (2009). Drought stress effects on Rubisco in wheat: changes in the Rubisco large subunit. Acta. Physiol. Plant..

[12] Rouphael Y., Cardarelli M., Schwarz D., Franken P., Colla G. Effects of drought on nutrient uptake and assimilation in vegetable crops. In: Aroca R, ed Plant responses to drought stress. Berlin Heidelberg, Germany: Springer, 171–195 (2012).

[13] Cramer MD, Hawkins HJ, Verboom GA (2009). The importance of nutritional regulation of plant water flux. Oecologia..

[14] Sardans J, Penuelas J (2012). The role of plants in the effects of global change on nutrient availability and stoichiometry in the plant-soil system. Plant. Physiol..

[15] Ackerson RC (1985). Osmoregulation in cotton in response to water-stress. 3. Effects of phosphorus fertility. Plant. Physiol..

[16] Premachandra GS, Saneoka H, Eujita K, Ogata SS (1990). Cell membrane stability and leaf water relations as affected by phosphorus nutrition under water stress in maize. Soil. Sci. Plant. Nutr..

[17] Faustino LI, Bulfe NML, Pinazo MA, Monteoliva SE, Graciano C (2013). Dry weight partitioning and hydraulic traits in young *Pinus taeda* trees fertilized with nitrogen and phosphorus in a subtropical area. Tree. Physiol..

[18] Campbell CD, Sage RF (2006). Interactions between the effects of atmospheric CO_2_ content and P nutrition on photosynthesis in white lupin (*Lupinus albus* L.). Plant Cell. Environ..

[19] Liu CG (2015). Effects of phosphorus application on photosynthetic carbon and nitrogen metabolism, water use efficiency and growth of dwarf bamboo (*Fargesia rufa*) subjected to water deficit. Plant Physiol. Biochem..

[20] dos Santos MG, Ribeiro RV, Oliveira RF, Pimentel C (2004). Gas exchange and yield response to foliar phosphorus application in *Phaseolus vulgaris* L. under drought. Braz. J. Plant Physiol..

[21] Liang WB (2010). A study of leaf tissue characteristic and drought resistance of *Alnus cremastogyne*. J. Central. South. University. For. Technol..

[22] Zhu WZ, Wang JX, Xue JH (2004). Water physiological characteristics of introduced *Alnus formosana*. J. Wuhan. Bot. Res..

[23] Hu H, Chen H, Hu T (2012). Adaptability comparison between the seedlings of *Eucalyptus grandis* and *Alnus cremastogyne* under the condition of continuous drought stress. J. Agric. Sci..

[24] Ge. T, Sun N, Bai L, Tong C, Sui F (2012). Effects of drought stress on phosphorus and potassium uptake dynamics in summer maize (*Zea mays*) throughout the growth cycle. Acta. Physiol. Plant..

[25] Rucker KS, Kvien CK, Holbrook CC, Hook JE (1995). Identification of peanut genotypes with improved drought avoidance traits. Peanut. Sci..

[26] Nonami H (1998). Plant water relations and control of cell elongation at low water potentials. J. Plant Res..

[27] Hu Y, Schmidhalter U (2001). Effects of salinity and macronutrient levels on micronutrients in wheat. J. Plant. Nutr..

[28] Graciano C, Guiamet JJ, Goya JF (2005). Impact of nitrogen and phosphorus fertilization on drought responses in *Eucalyptus grandis* seedlings. For. Ecol. Manag..

[29] Jones CA, Jacobsen JS, Wraithl JM (2005). Response of malt barley to phosphorus fertilization under drought conditions. J. Plant. Nutr.

[30] Oliveira MT, Medeiros CD, Frosi G, dos Santos MG (2014). Different mechanisms drive the performance of native and invasive woody species in response to leaf phosphorus supply during periods of drought stress and recovery. Plant. Physiol. Biochem..

[31] Liu C (2011). Effect of drought on pigments, osmotic adjustment and antioxidant enzymes in six woody plant species in karst habitats of southwestern China. Environ. Exp. Bot..

[32] Yang F, Miao LF (2010). Adaptive responses to progressive drought stress in two poplar species originating from different altitudes. Silva. Fennica..

[33] Shubhra D, Goswami J (2004). C. L., Munjal, R. Influence of phosphorus application on water relations, biochemical parameters and gum content in cluster bean under water deficit. Biol. Plant..

[34] Garg BK, Burman U, Kathju S (2004). The influence of phosphorus nutrition on the physiological response of moth bean genotypes to drought. J. Plant Nutr. Soil. Sci..

[35] Sauter A, Davies WJ, Hartung W (2001). The long distance abscisic acid signal in the droughted plant: the fate of the hormone on its way from root to shoot. J. Exp. Bot..

[36] Tezara W, Mitchell VJ, Driscoll SD, Lawlor DW (1999). Water stress inhibits plant photosynthesis by decreasing coupling factor and ATP. Nature..

[37] Singh SK, Badgujar G, Reddy VR, Fleisher DH, Bunce JA (2013). Carbon dioxide diffusion across stomata and mesophyll and photo-biochemical processes as affected by growth CO_2_ and phosphorus nutrition in cotton. J. Plant. Physiol..

[38] Zhang J, Kirkham MB (1996). Antioxidant response to drought in sunflower and sorghum seedlings. New. Phytol..

[39] Frosi G, Oliveira MT, Almeida-Cortez JS, dos Santos MG (2013). Ecophysiological performance of *Calotropis procera*: an exotic and evergreen species in Caatinga, Brazilian semi-arid. Acta. Physiol. Plant..

[40] Rivas R, Oliveira MT, dos Santos MG (2013). Three cycles of water deficit from seed to young plants of *Moringa oleifera* woody species improves stress tolerance. Plant Physiol. Biochem..

[41] Wang L (2014). Physiological and molecular responses to drought stress in rubber tree (Hevea brasiliensis Muell. Arg.). Plant Physiol. Biochem..

[42] Carvalho MHCD (2008). Drought stress and reactive oxygen species. Plant. Signal. Behav..

[43] Keunen E, Peshev D, Vangronsveld J, Ende WVD, Cuypers A (2013). Plant sugars are crucial players in the oxidative challenge during abiotic stress: extending the traditional concept. Plant. Cell. Environ..

[44] Al-Karaki GN, Clark RB, Sullivan CY (1996). Phosphorous nutrition and water stress effects on proline accumulation in sorghum and bean. J. Plant. Physiol..

[45] Azcon R, Gomez M, Tobar R (1996). Physiological and nutritional responses by *Lactuca sativa* L. to nitrogen sources and mychorrhizal fungi under drought condition. Biol. Fert. Soil..

[46] Olsen, S. R., Sommers, L. E. Phosphorus, in: A. L. Page, R. H. Miller, D. R. Keeney (Eds), Methods of Soil Analysis, Part 2, Agronomy Society of America and Soil Science Society of America, 1982, pp. 403–430. Madison, WI (1982).

[47] Murphy J, Riley JP (1962). A modified single solution method for the determination of phosphate in natural waters. Anal. Chim. Acta..

[48] Xu ZZ, Zhou GS, Shimizu H (2009). Are plant growth and photosynthesis limited by pre-drought following rewatering in grass?. J. Exp. Bot..

[49] Liu CG (2014). Carbon and nitrogen metabolism in leaves and roots of dwarf bamboo (*Fargesia denudata* Yi) subjected to drought for two consecutive years during sprouting period. J. Plant Growth Regul..

[50] Zhang, Z. L., Qu, W. J. The Experimental Guidance of Plant Physiology. Higher Education Press, Beijing, pp. 127–137 (2003).

[51] Elstner EF, Heupel A (1976). Formation of hydrogen peroxide by isolated cell walls from horseradish (*Armoracia lapathifolia* Gilib.). Planta..

[52] Patterson BD, MacRae EA, Ferguson IB (1984). Estimation of hydrogen peroxide in plant extracts using titanium (IV). Anal. Biochem..

[53] Zhou Y, Lam HM, Zhang J (2007). Inhibition of photosynthesis and energy dissipation induced by water and high light stresses in rice. J. Exp. Bot..

[54] Fu J, Huang B (2001). Involvement of antioxidants and lipid peroxidation in the adaptation of two cool-season grasses to localized drought stress. Environ. Exp. Bot..

